# Rethinking causal inference for recurring exposures: The incremental propensity score approach with lavaan

**DOI:** 10.3758/s13428-025-02735-x

**Published:** 2025-07-18

**Authors:** Wen Wei Loh, Dongning Ren, Yves Rosseel

**Affiliations:** 1 Department of Methodology and Statistics, Faculty of Health, Medicine and Life Sciences (FHML), Maastricht University, Postbus 616, 6200 MD Maastricht, The Netherlands; 2 Department of Work and Social Psychology, Maastricht University, Maastricht, The Netherlands; 3 Department of Data Analysis, Ghent University, Ghent, Belgium

**Keywords:** Causal inference, Incremental propensity score intervention (IPSI), Longitudinal data, Potential outcomes, Time-varying or treatment-dependent confounding

## Abstract

Scholars are often interested in evaluating the causal effects of a recurring exposure (e.g., family violence) on behavioral and psychological outcomes. However, causal inference of recurring exposures is challenging. Conventional analytic approaches target causal quantities lacking practical relevance, such as mandating everyone to uniformly always be exposed or unexposed to family violence. Estimation further relies on everyone having a non-zero probability of being either exposed or unexposed at each occurrence, which is frequently unrealistic when past exposures perfectly predict future exposures. In this paper, we introduce a novel approach from the causal inference literature for drawing causal conclusions about recurring exposures: the incremental propensity score intervention (IPSI). IPSI frames causal questions more realistically by assessing how changing the propensity of a recurring exposure may influence an outcome. To facilitate the adoption of IPSI for recurring exposures, we develop an estimation procedure using lavaan, a widely used structural equation modeling software in R. We demonstrate the application of IPSI with a real-world dataset investigating the impact of recurring family violence on adolescent depression. IPSI requires fewer assumptions than existing approaches while offering more meaningful insights into the causal effects of recurring exposures.

In the psychological and behavioral sciences, researchers often seek to evaluate the causal effects of exposures that occur repeatedly. Examples include, but are not limited to, individual behaviors such as social media use (Valkenburg, 2022) and socializing (Ren et al., 2022); lifestyle factors such as physical activity (Smith & Merwin, 2021) and sleep quality (Scott et al., 2021); and social stressors such as school bullying (Hymel & Swearer, 2015) and workplace aggression (Zhong et al., 2023). Common to these exposures is that they rarely occur only once. In reality, such exposures occur intermittently or periodically on multiple occasions and are innately *recurring*.

Various methods have been developed to assess the causal effects of recurring exposures. These include marginal structural models using inverse probability weighting (Cole & Hernán, 2008; Robins et al., 2000), parametric g-formula (Robins, 1986; Westreich et al., 2012), and structural nested mean models using g-estimation (Robins, 1994; Vansteelandt & Sjölander, 2016). These methods are widely applied in epidemiology and medical sciences (Clare et al., 2019; Mansournia et al., 2017) and are gaining traction in psychological and behavioral sciences (Kennedy et al., 2022; Thoemmes & Ong, 2015). These methods have recently been introduced to the psychological and behavioral research literature, such as g-estimation (Loh & Ren, 2023a) and parametric g-formula (Loh et al., 2024).

Common to these methods is their focus on causal effects comparing counterfactual scenarios where at each time point, everyone uniformly experiences the same exposure (Bonvini et al., 2023). While such effects may be precisely what researchers intend in specific contexts – for example, taking vs. not taking a prophylactic medication daily – their practical relevance can be limited in behavioral research. This is especially true when it is unrealistic for everyone’s exposure to be identical, such as the examples we provided above: social media use, socializing, and being the victim of bullying or aggression, among others. Therefore, while these methods are valuable tools for causal inference, their applicability in behavioral research warrants careful consideration of the context and implications of the causal effects.

In this article, we put forth a novel approach – unencumbered by this drawback – to evaluate the causal effects of recurring exposures: the *incremental propensity score intervention* (IPSI; Kennedy (2019)). IPSI frames causal queries using a realistic and practical question: How would changing the chances of a recurring exposure by a given amount lead to different average outcomes? IPSI has been applied in criminology to investigate the effect of homelessness on recidivism (Jacobs et al., 2024), and in epidemiology to investigate the effects of consuming vegetables on preeclampsia (Naimi et al., 2021) and of aspirin on the incidence of pregnancy (Rudolph et al., 2022). Loh and Ren (2024) offer an accessible introduction to IPSIs with a time-fixed exposure (i.e., measured at a single time point) for a behavioral sciences audience.

Our paper proceeds in three parts. First, we describe what the IPSI for recurring exposures is, and why this approach offers realistic and meaningful evaluations of the causal effects of recurring exposures. Second, we present the causal conditions that suffice to estimate the IPSI. Crucially, to make IPSI accessible to a broad audience of behavioral and psychological researchers, we implement the procedure using *lavaan* (Rosseel, 2012), a widely used and freely accessible structural equation modeling software in R (R Core Team, 2021). Finally, we illustrate IPSI for recurring exposures using a publicly available real-world dataset. To enhance readers’ understanding of IPSI, throughout the paper, we use a running example from psychological research: the causal effect of recurring family violence on depression. We explain how IPSI offers unique insights into the accumulative impact of exposure to family conflicts over time on the development of depression among adolescents. All the R scripts implementing the procedure are freely available online (https://osf.io/8ksfx/).

## Using the IPSI to assess causal effects of recurring exposures

We will use a running example from psychological research to ease the exposition of IPSI. Suppose researchers are investigating the causal effect of family violence. Family violence is a recurring exposure because it occurs repeatedly on multiple occasions. Suppose the research interest is in whether reducing family violence (binary exposure $X_t$) at consecutive time points $t=1,\ldots ,T$ leads to lower depression (outcome *Y*) among adolescents. (To simplify our introduction of the IPSI, we first consider a single end-of-study outcome, and then generalize to repeatedly measured outcomes later.) The researchers seek to answer the causal query: How would depression change, on average, among adolescents if the chances of family violence over time could be hypothetically lowered?

To answer this question, we formally define what it means to lower the probability of family violence. Let $\pi _t\equiv {{\,\textrm{Prob}\,}}(X_t=1|H_t)$ denote an individual’s *propensity score* (Rosenbaum & Rubin, 1983b; Thoemmes & Ong, 2015; West et al., 2014) at time *t*, which is the conditional probability of being exposed to family violence $(X_t)$ given pre-exposure covariates $H_t$ (we elaborate on $H_t$ later). Now, suppose the researchers can *shift* the propensity of family violence from the naturally occurring quantity $\pi _t$ to a lower probability instead, e.g., through a hypothetical education program or legislative policy to prevent or mitigate domestic violence. Denote this shifted probability by $Q_t^\delta \equiv Q_t^\delta (X_t=1|H_t)$, which can be expressed in terms of $\pi _t$ as:

1 $$\begin{aligned} Q_t^\delta = \dfrac{\delta \pi _t}{1 + (\delta -1)\pi _t}. \end{aligned}$$

This specific form was used because solving for $\delta$ yields the relationship:

2 $$\begin{aligned} \delta = \dfrac{Q_{t}^{\delta }}{1-Q_{t}^{\delta }} \big / \dfrac{\pi _{t}}{1-\pi _{t}} . . \end{aligned}$$

Hence, $\delta$, termed a *shift parameter*, encodes the odds ratio between the shifted propensity score $Q_t^\delta$ and the naturally occurring propensity score $\pi _t$. In other words, at time *t*, researchers can posit a value of $\delta$, then multiply it with the odds for the (naturally occurring) propensity score to obtain the shifted odds in (1) for each individual. For example, when $\delta <1$, the shifted risk of family violence will be lower than the naturally occurring risk. We emphasize that while the exposure (family violence) is binary, the propensity for it to occur is distributed along a continuum. In general, $Q_t^\delta$, hereafter referred to simply as IPSI, is termed an *incremental propensity score intervention* (Kennedy, 2019) because it characterizes the shifted propensity score following a hypothetical intervention that changes the naturally occurring odds by $\delta$.

The accumulation of a recurring exposure over time can affect later outcomes that cannot be captured by a snapshot at a single time. Therefore, the causal query focuses on whether shifting the propensity for a recurring exposure throughout the population can change the average outcome. Continuing our example, researchers can conceive of hypothetically lowering the recurring propensity of family violence by a given amount and then evaluate the average potential depression following this reduction. Denote the IPSI for the recurring exposure under a posited $\delta$ by ${\varvec{Q}}^\delta = \left\{ Q_t^\delta : t=1,\ldots ,T\right\}$. Denote the potential outcome^1^ (Rubin, 1990; Splawa-Neyman et al., 1990) an individual would have had, possibly counter to fact, after experiencing the recurring exposures $(X_1, \ldots , X_T)=(x_1,\ldots ,x_T)$ by $Y^{x_1, \ldots , x_T}$.

For example, the potential depression following recurring family violence at $T=2$ time points is $Y^{x_1, x_2}$. We can assess the impact of an IPSI ${\varvec{Q}}^\delta = \left\{ Q_1^\delta ,Q_2^\delta \right\}$ on the average depression using the following causal quantity:

3 $$\begin{aligned} {{\,\textrm{E}\,}}(Y^{{\varvec{Q}}^\delta })&\equiv \sum _{c \in {\mathcal {C}}} \sum _{l_1 \in {\mathcal {L}}_1} \sum _{l_2 \in {\mathcal {L}}_2} \sum _{x_1=0}^{1} \sum _{x_2=0}^{1} {{\,\textrm{E}\,}}(Y^{x_1, x_2} | h_2, x_2) \nonumber \\&\quad \times Q_1^\delta (x_1|h_1) Q_2^\delta (x_2|h_2) {{\,\textrm{p}\,}}(l_2 | h_{1}, x_{1}) {{\,\textrm{p}\,}}(l_1 | c). \end{aligned}$$

Here, ${{\,\textrm{E}\,}}(A|b)$ and ${{\,\textrm{p}\,}}(a|b)$ denote the conditional expectation and probability function of a random variable *A* among those with a specific value(s) of the random variable(s) $B=b$, respectively. Uppercase letters represent random variables or potential outcomes, while lowercase letters indicate specific or realized values. The support for the time-invariant (*C*) and the time-varying ($L_t$) covariates are denoted by $\mathcal {C}$ and ${\mathcal {L}}_t$, respectively, and are assumed to be discrete. When a covariate is continuous, the summation and probability mass function may be replaced by an integral and probability density function, respectively; see Kennedy (2019) for more general settings.

In words, the IPSI causal estimand in (3), hereafter referred to as IPSICE for simplicity, is a cumulative weighted average of the potential outcomes over the recurring exposure, with weights at each time corresponding to the cumulative product of IPSI (up to that time) under given $\delta$. Continuing our example, the causal quantity in (3) can be interpreted as the average potential depression when each individual’s odds of family violence at both times are multiplied by $\delta$. Put another way, the causal quantity in (3) answers the questions: Would raising the propensity of being exposed to recurring family violence engender a subsequent increase in depression? Conversely, would reducing the propensity of being exposed to recurring family violence engender a subsequent decrease in depression?

More generally, the average potential outcome under an IPSI ${\varvec{Q}}^\delta$ is:

4 $$\begin{aligned} {{\,\textrm{E}\,}}(Y^{{\varvec{Q}}^\delta })&\!\equiv \! \sum _{c \in {\mathcal {C}}} \sum _{l_1 \in {\mathcal {L}}_1} \!\cdots \! \sum _{l_T \in {\mathcal {L}}_T} \sum _{x_1=0}^{1} \cdots \sum _{x_T=0}^{1}\! {{\,\textrm{E}\,}}(Y^{x_1, \!\ldots ,\! x_T} | h_T, x_T)\nonumber \\&\quad \prod _{t=1}^{T} Q_t^\delta (x_t|h_t) {{\,\textrm{p}\,}}(l_t | h_{t-1}, x_{t-1}). \end{aligned}$$

Continuing our example, the causal quantity in (4) can be interpreted as the average potential depression when each individual’s odds of family violence at all times are multiplied by $\delta$. Hence, setting $T=3$ in (4) renders the causal quantity in (3). Note that when $\delta =1$, the IPSI reverts to the naturally occurring PS (i.e., $Q_t^\delta = \pi _t$) and (4) is just the average observed outcome.

We briefly compare IPSICE with prevailing *average causal effects* (ACEs; Rubin (1974)) that are (implicitly) targeted in conventional analyses. First, an ACE necessarily compares different counterfactual scenarios mandating everyone at the same exposure level(s). Continuing our example, an ACE may compare the average potential outcomes where, throughout the study, everyone is subject to family violence vs. no one is subject to family violence. Such extreme, uniform scenarios are untethered from reality because neither corresponds to what was actually observed. Recurring exposures, such as family violence, are intrinsically heterogeneous, with some individuals being exposed periodically while others might be exposed only sporadically.

Moreover, because interventions that can realistically achieve such counterfactual scenarios are rarely feasible at best or impossible at worst, the ACE’s interpretation has limited or no practical implications. In contrast, IPSICE integrates individual heterogeneity in the propensity of being exposed to family violence in its definition, and its interpretation is characterized by shifting each individual’s propensity score.

Second, everyone must have a non-zero probability of being exposed or not exposed to family violence at each time *t*; i.e., $0<\pi _t<1$ for all $t=1,\ldots ,T$. This so-called *positivity* assumption (Westreich & Cole, 2010), is violated when certain combinations of the pre-exposure variables perfectly predict the exposure (e.g., producing the problem of complete or perfect separation when fitting a logistic regression model for the propensity score). This is particularly salient for recurring exposures in behavioral research because, frequently in naturalistic real-world settings, earlier exposures overwhelmingly influence future exposure. Continuing our example, prior exposure to family violence can systematically induce vulnerability to subsequent occurrences (e.g., all individuals with $X_{1}=1$ have a predicted probability of $X_2=1$ being precisely one). Violations of the positivity assumption are challenging to diagnose, but they can result in extrapolation for the ACE and potentially biased estimates at best and ill-defined effects in non-existent covariate strata at worst (Rudolph et al., 2022). In contrast, IPSI is not bound by this assumption because, for those whose probability of (non-)exposure is zero, their potential outcomes do not contribute to the average outcome.

The average potential outcomes defined in (3) and (4) are identifiable under certain assumptions common to almost all causal inferences from observational data. The first is causal or counterfactual consistency, stated as Assumption 1 of Kennedy (2019), which assumes that the observed treatments $(X_1, \ldots , X_T)$ reveal the potential outcome as the observed outcome; i.e., $Y=Y^{X_1, \ldots , X_T}$. The second is no unmeasured confounding (Imbens & Rubin, 2015), where within strata defined by the unique values of the pre-exposure variables recorded in $H_t$, the exposure $X_t$ and potential outcomes $Y^{x_1, \ldots , x_T}$ are conditionally independent. This can be formally stated, following Assumption 2 of Kennedy (2019), as:

5 $$\begin{aligned} Y^{x_{1}, \ldots , x_{T}} \perp \!\!\!\perp X_{t} | H_{t}, \end{aligned}$$

for all possible values $x_1, \ldots , x_T$, where $A \perp \!\!\!\perp B | C$ denotes the conditional independence of *A* and *B* given *C*. When both Assumptions 1 and 2 of Kennedy (2019) are met, the causal quantity ${{\,\textrm{E}\,}}(Y^{x_1, \ldots , x_T}|h_T,x_T)={{\,\textrm{E}\,}}(Y|h_T,x_T)$ can be consistently estimated from observed non-causal data.

Because $X_t$ is a recurring exposure, the variables in $H_t$ causally preceding $X_t$ include time-invariant covariates (*C*), past exposures ($X_1, \ldots , X_{t-1}$) and the time-varying covariate history ($L_1, \ldots , L_t$). Therefore, $H_t = (C, L_1, X_1, \ldots , L_{t-1}, X_{t-1}, L_t)$. In practice, outcomes are also repeatedly measured over time in longitudinal designs with recurring exposures. With repeatedly measured outcomes, the definitions and results above readily extend by considering the outcome at each time point as the final outcome one at a time. However, $H_t$ now includes the outcome and exposure history (Loh & Ren, 2023b), as well as the pre-exposure covariates. For example, if within each time point *t*, $X_t$ causally precedes $Y_t$, then $H_t = (C, L_1, X_1, Y_1, \ldots , L_{t-1}, X_{t-1}, Y_{t-1}, L_t)$. Alternatively, if within each time point *t*, $Y_t$ causally precedes $X_t$, then $H_t = (C, L_1, Y_1, X_1, \ldots , L_{t-1}, Y_{t-1}, X_{t-1}, L_t, Y_t)$.

Consistent estimation of the IPSI causal quantities is contingent on no unmeasured confounding (5) being met. Although no sensitivity analyses specific to IPSI are currently available, existing methods already developed for other causal effect estimands may be extended for IPSI. One promising possibility is to adapt the sensitivity analysis of Bonvini and Kennedy (2022) by permitting a proportion of the population to be subject to unmeasured confounding and then reanalyzing the data under that scenario. Here, we propose another approach previously put forth in other contexts (Harring et al., 2017; Loh, 2024) that is amenable to use in lavaan. Because unmeasured confounding manifests in correlated exposure and outcome residuals, we can encode their correlation as a sensitivity parameter (Lin et al., 1998; Rosenbaum & Rubin, 1983a). Stronger correlations indicate more severe violations of unmeasured confounding, whereas a zero correlation corresponds to no violations. Hence, we leverage lavaan to mimic unmeasured confounding using correlated residuals as follows: First, fix the correlation between each exposure and a causally downstream outcome at a given value as part of the lavaan model. (Residual covariances are readily specified in lavaan using two adjoining tilde symbols, i.e., “$\sim \sim$”.) Next, fit the model with this fixed correlation to the observed data and estimate the IPSICE under this constraint. Repeating both steps using different fixed values of the sensitivity parameter permits systematically judging the extent to which unmeasured confounding alters the results. We illustrate its implementation using real-world data in the next section.

**Table 1:** Observed data for six students in the illustration

ID	Gender	Race	Age	Income	$L_1$	$X_1$	$Y_1$	$L_2$	$X_2$	$Y_2$	$L_3$	$X_3$	$Y_3$
8	1	1	1	1	1.12	1	1.40	0.26	1	1.40	-1.37	1	1.20
16	0	0	0	1	0.46	1	1.00	1.51	1	1.20	0.38	1	1.00
24	1	1	0	1	-1.21	0	1.00	0.76	0	1.60	-1.12	1	3.40
32	0	0	0	1	-0.54	1	1.00	-0.74	1	1.40	0.30	1	1.80
40	0	1	1	1	0.04	1	1.40	0.76	1	3.20	-0.37	1	1.60
48	1	1	0	0	0.54	1	3.20	-1.24	1	1.20	0.63	1	3.20

*Note*. “Income” = family socioeconomic status; $X_t=$ whether family violence occurred at least once in a while (1) or hardly ever occurred (0) at time *t*; $L_t=$ stress at time *t* (mean-centered); $Y_t=$ recent depression at time *t*. The definitions of each variable are in the main text. Values for continuous $L_t$ and $Y_t$ were rounded to two decimal places

**Fig. 1:**
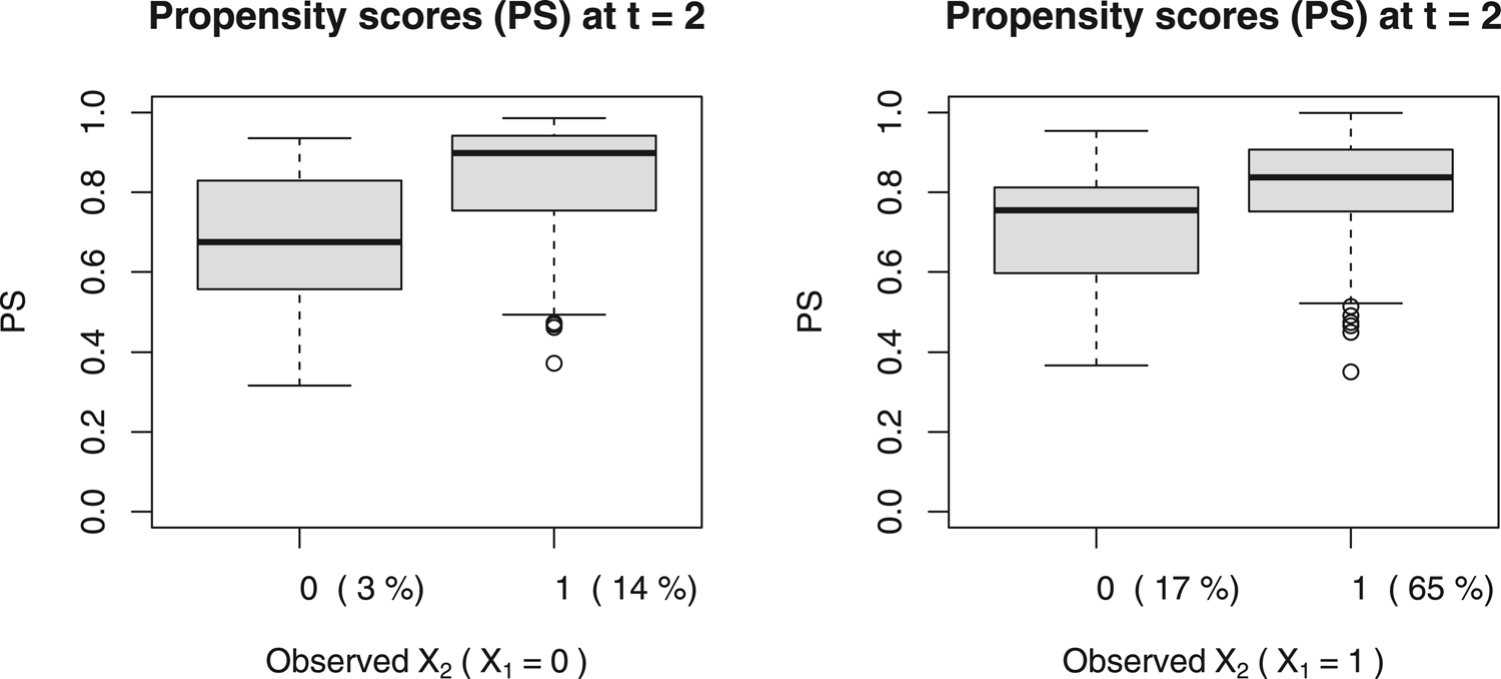
Estimates of the naturally occurring propensity scores at $t=2$ for each exposure group, stratified by the previous exposure $X_1$, in the family violence example. The proportions of those observed with either $X_2=0$ or $X_2=1$ across strata defined by $X_1$ are stated in brackets on the horizontal axis

## Illustration using real-world data

We illustrate IPSI using data from a real-world study: the Flint Adolescent Study. This longitudinal study, beginning in 1994, followed ninth graders from four public high schools in Flint, Michigan (Zimmerman, 2014). For illustration, we will use data from three study waves (hereafter time points $t=1,2,3$) to investigate whether reducing family violence leads to decreased depression over time. Both variables were repeatedly recorded at each time point.

We utilized three waves of the survey: $1995 \, (t=1), 1996 \, (t=2)$, and $1997 \, (t=3)$. The exposure $X_t$ (family violence) was a composite of five dichotomous variables measuring students’ responses to whether the following family situations occurred at least once in a while or hardly ever: (i) “We fight in our family,” (ii) “Family members get so angry they throw things,” (iii) “Family members lose their tempers,” (iv) “Family members criticize each other,” and (v) “Family members hit each other in anger.” The exposure took on a value of $X_t=1$ if any situation occurred at least once in a while around time *t* or a value of $X_t=0$ if all these situations hardly ever occurred around time *t*. The outcome $Y_t$ (depression) was measured using the mean score for five items on how much the student felt uncomfortable because of the following problems during the past week (including the day of the interview): (i) “had thoughts of ending your life,” (ii) “felt blue,” (iii) “felt no interest in things,” (iv) “felt hopeless about the future,” and (v) “felt worthless.” Responses were on a five-point Likert scale (1=Not at all uncomfortable; 5=Extremely uncomfortable).

To simplify our illustration, we considered a single time-varying covariate $L_t$: stress levels, measured using the mean score of four items on how much stress they felt in different areas of their life (at school, at work, with friends, with personal issues or concerns) during the past 6 months. Responses were on a five-point Likert scale (1=Very low stress; 5=Very high stress). The baseline covariates *C* were the following self-reported variables: age cohort (1 if their birth year was 1980, 0 if earlier than 1980), gender (1 for female, 0 for male), race (1 for Black or African American, 0 otherwise), and family socioeconomic status (1 if their family received income support, 0 otherwise). A snapshot of the data for six students is presented in Table 1. To simplify the illustration, we considered the 753 students with complete data on these variables.

Ninety percent of the students were subject to repeated family violence (in at least two time points), with 49% being subject throughout the study. This emphasizes that exposure to family violence was innately recurring, with each additional occurrence adding to the cascade of future occurrences. Crucially, the recurring exposures to family violence are so tightly interlinked that isolating the effect at a single time point would be artificial and pointless.

**Fig. 2:**
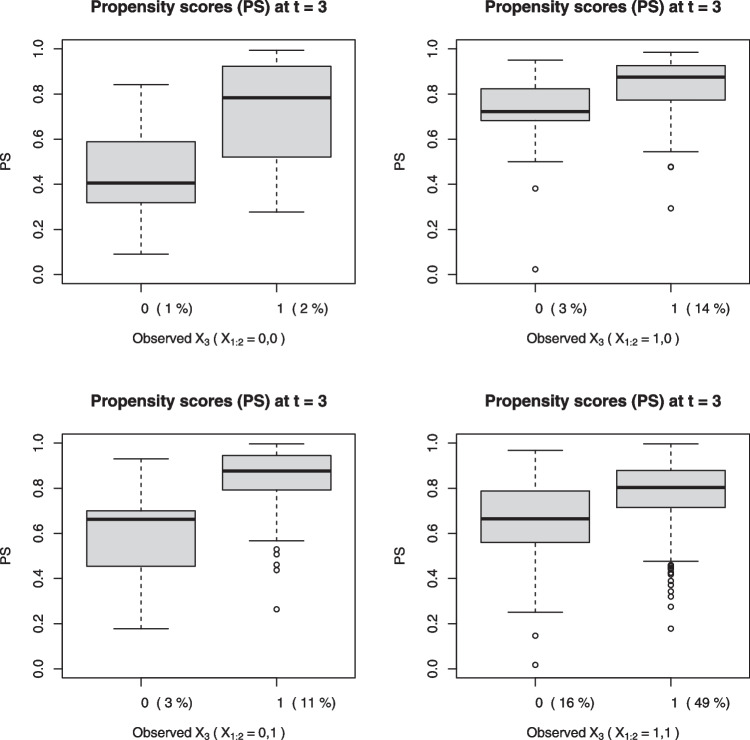
Estimates of the naturally occurring propensity score at $t=3$ for each exposure group, stratified by the previous exposures $X_1$ and $X_2$, in the family violence example. *Note*. $X_{1:2}=(X_1, X_2)$. The proportions of those observed with either $X_3=0$ or $X_3=1$ across strata defined by $(X_1, X_2)$ are stated in brackets on the horizontal axis

To check whether positivity was empirically violated, we plotted the predicted propensity scores for both exposure groups ($X_t=0$ and $X_t=1$) at each time $t=2$ and $t=3$, in Figs. 1 and 2, respectively. We stratified the propensity scores based on previous exposure(s) to better illustrate the recurring nature of family violence. Note how some students exposed to family violence ($X_t=1$) had propensity scores very close to one, more so than those unexposed to family violence ($X_t=0$) who tended to have propensity scores closer to zero. The lack of overlap in interquartile ranges between the two exposure groups was most pronounced at $t=3$ among those previously exposed to family violence but not earlier ($X_2=1,X_1=0$); see the bottom left panel of Fig. 2.

**Fig. 3:**
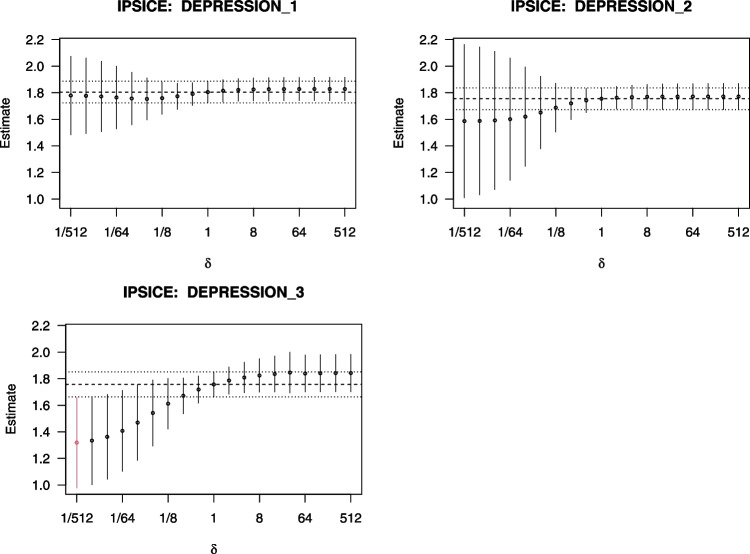
Estimates of the incremental propensity score intervention causal estimand (IPSICE) for the outcome at each time point in the family violence example. *Note.* Each *circle* corresponds to an IPSICE estimate, and each *vertical line* corresponds to the 95% confidence interval (CI), for a given value of $\delta$ on the horizontal axis. A logarithmic horizontal axis was used for the evenly spaced posited values of $\delta$ on a binary logarithm scale. The *horizontal dashed* and *dotted lines* corresponded to the average observed outcome and 95% CI, respectively, under $\delta =1$. Values of $\delta$ whose CIs did not overlap the latter were indicated in red

We carried out the estimation procedure described in Appendix A. Briefly, for the propensity scores $\pi _t$, we fitted logistic models with main effects and two-way interaction terms for all the predictors in $H_t$. An example of the specified logistic regression model for $X_1$ is formally stated in (A.1) of the Appendix. For the outcomes $Y_t$, we fitted regression models with main effects for $(X_t, H_t)$ and two-way interaction terms between each exposure instance $X_s,s=1,\ldots ,t$, and each variable in $H_t$ excluding that exposure, i.e., $H_t \setminus X_s$. Examples of the specified regression models for $Y_1$ and $Y_2$ are formally stated in (A.4) and (A.5), respectively, of the Appendix. The regression models for repeated outcomes were fitted jointly using lavaan. For the IPSI, we posited a discrete grid of $\Delta =\{2^{-9}, 2^{-8}, \ldots , 2^{9}\}$. Estimates of the average outcomes at each of the three time points in terms of $\delta$ are displayed in Fig. 3.

There was no effect of family violence on average depression in the first two times. However, there was an effect of recurring family violence on average depression at the third time point. Multiplying the odds of family violence by a factor of $\delta =2^{-9}=1/512$ at all three times reduced subsequent average observed depression by 0.44 points on a five-point scale. We further illustrated a sensitivity analysis of unmeasured confounding for IPSI, leveraging lavaan as described in the preceding section. To simplify the analysis, we set the correlations between the residuals for family violence and depression to be greater than 0.3, allowing for mild to strong residual confounding. The results are plotted in Fig. 5 of the Appendix, and suggest that the odds of family violence must be lowered over time to reduce average depression, albeit not statistically significantly, even after accounting for unmeasured confounding. There is no single time point at which family violence can be shifted to reduce average depression; only when the full array of the recurring exposure is shifted can average depression be reduced. These results suggested that to reduce average depression among adolescents, it is inadequate to merely lower family violence at a single time point because enduring effort is required for its impact to accumulate over time and emerge.

## Discussion

In this paper, we introduced the IPSICE for recurring exposures. We implemented an estimator that leverages lavaan, a software widely used by psychological and behavioral researchers.^2^ A major advantage of using lavaan is access to its various features when fitting a set of outcome models jointly (see step 4a of the estimation procedure in the Appendix). We describe two examples relevant to IPSICE. First, parameter constraints can be easily introduced in the lavaan model syntax. For example, the effect of $X_t$ on $Y_{t}$ can be readily constrained to be constant across times *t*; similarly, the autoregressive effects of $Y_{t-1}$ on $Y_t$ can also be constrained to be stable over time. Second, intermittently missing data can be straightforwardly handled within lavaan. For example, “full information” maximum likelihood estimation (Lee & Shi, 2021) can be readily utilized (by using the argument missing = “ML”) after establishing the missing mechanism is missing at random (MAR; Yuan and Bentler (2010)).

**Fig. 4:**
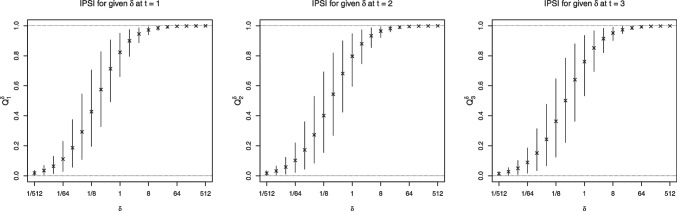
Estimates of the IPSIs $Q_t^\delta$ at $t=1,2,3$ in the family violence example. *Note*. For a given value of $\delta$ on the horizontal axis, the estimated average ${{\,\textrm{E}\,}}[Q_t^\delta ]$ is denoted by a cross, with each vertical line corresponding to a symmetric 80-percentile interval. A logarithmic horizontal axis was used for the evenly spaced posited values of $\delta$ on a binary logarithm scale

In practice, the posited values of $\delta$ should be based on prior empirical results, established theory, or policy guidelines or recommendations. We offer two practical suggestions. First, researchers should consider a broad range of values to produce IPSIs with substantively different magnitudes. Second, researchers should be aware that very small or large values can lead to individual chances of exposure being close to zero or one, respectively. This may produce counterfactual scenarios where almost everyone is unexposed or exposed. To illustrate this point, we plotted the IPSIs for the family violence example in Fig. 4. Note now at $\delta =1/512$, the average propensity score was close to zero; under this IPSI, only about 4–6% of individuals had chances of being exposed to family violence above 0.05. Therefore, researchers should use their domain or subject matter knowledge to evaluate whether or not such scenarios are realistic in the specific context of the exposure.

### Conclusion

In conclusion, the IPSI approach is ideally suited for evaluating the causal effects of recurring exposures. By effectively adopting the IPSI in empirical analyses, we seek to contribute to more realistic and meaningful assessments of recurring exposures in psychological and behavioral research.

## Figures and Tables

**Fig. 5:**
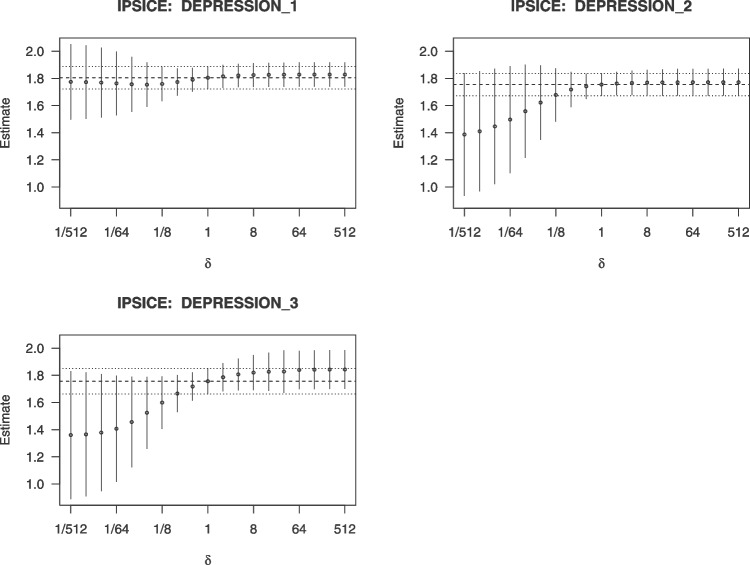
Estimates of the incremental propensity score intervention causal estimand (IPSICE) for the outcome at each time point in the family violence example, after using correlated exposure and outcome residuals to mimic unmeasured confounding. *Note*. Descriptions of each panel are provided as part of Fig. 3 in the main text

## Data Availability

The data used in the illustration is publicly available at https://doi.org/10.3886/ICPSR34598.v1.

## Appendix A: Estimating the IPSICE for recurring exposures

In this section, we describe how to empirically estimate the IPSICEs using observed data. The estimation procedure extends the algorithm in Web Appendix Section 1.3 of Kennedy (2019) for a single outcome to handle repeatedly measured outcomes simultaneously.

1. For each exposure time point $t=1,\ldots ,T$, in turn, fit a model for the naturally occurring propensity score $\pi _t$ to the observed data. A simple example of a propensity score model is a logistic regression model for $X_t$ given main effects and two-way interaction terms for the observed pre-exposure history in $H_t$; e.g., for $t=1$ and $H_1=(C,L_1)$,
  A.1 $$\begin{aligned} {{\,\textrm{logit}\,}}\left\{ \Pr (X_1 =1 | H_1)\right\} = \alpha _{1,0} + \alpha _{1,1} L_1 + \alpha _{1,2} C + \alpha _{1,3} CL_1. \end{aligned}$$
  The model in (A.1) may be fitted using, e.g., maximum likelihood estimation. Then, using the fitted model, obtain the predicted propensity score at time *t* for each individual, denoted by $\hat{\pi }_t$ for simplicity. The logistic models specified in the illustration are included as R model formulae in the online supplemental materials.
2. Calculate the weights for all exposure time points $t=1,\ldots ,T$ for a given $\delta$:
  A.2 $$\begin{aligned} W_t \!&=\! \dfrac{1+(\delta -1)X_t}{1+(\delta -1)\hat{\pi }_t},&V_t \!&=\! \dfrac{X_t(1-\hat{\pi }_t)\!-\!\delta (1-X_t)\hat{\pi }_t}{\delta /(\delta -1)}. \end{aligned}$$
3. Calculate the cumulative weight for $t=1,\ldots ,T$:
  A.3 $$\begin{aligned} \tilde{W}_t = \prod _{s=1}^{t} W_s. \end{aligned}$$
4. We now describe how to parsimoniously fit a joint model simultaneously for the repeated outcomes at all time points by leveraging lavaan (Rosseel, 2012). For notational simplicity, let $Y_{t^\prime }$ denote the outcome at the first time point $t^\prime$ immediately following the exposure at time *t*. If within each time point *t*, $X_t$ causally precedes $Y_t$, then set $t^\prime = t$; otherwise if within each time point *t*, $Y_t$ causally precedes $X_t$, then set $t^\prime = t+1$. For $d=0,\ldots ,T-1$:
  - Regress the (pseudo-)outcomes $R_{t^\prime +d, t+1}$ on $X_t$ and $H_t$ for $t=1,\ldots ,T-d$. When $d=0$, use the observed outcomes $R_{t^\prime , t+1} \equiv Y_{t^\prime }$. For example, regression models with main effects for $(X_t, H_t)$ and two-way interaction terms between each exposure instance $X_s,s=1,\ldots ,t$, and each variable in $H_t \setminus X_s$, for $d=0, T=2, t^\prime =t$, are:
    A.4 $$\begin{aligned} {{\,\textrm{E}\,}}(Y_1 | X_1, H_1)&= \beta _{1,0} + \beta _{1,1}X_1 + \beta _{1,2}L_1 \nonumber \\&\quad + \beta _{1,3}C+ \beta _{1,4}X_1L_1 + \beta _{1,5}X_1C; \end{aligned}$$
    A.5 $$\begin{aligned} {{\,\textrm{E}\,}}(Y_2 | X_2, H_2)&= \beta _{2,0} + \beta _{2,1}X_2 + \beta _{2,2}L_2 + \beta _{2,3}X_1 \nonumber \\&\quad + \beta _{2,4}Y_1 + \beta _{2,5}L_1 + \beta _{2,6}C \nonumber \\&\quad + \beta _{2,7}X_2L_2 + \beta _{2,8}X_2X_1 \nonumber \\&\quad + \beta _{2,9}X_2Y_1 + \beta _{2,10}X_2L_1 \nonumber \\&\quad + \beta _{2,11}X_2C + \beta _{2,12}X_1L_2 \nonumber \\&\quad + \beta _{2,13}X_1Y_1 + \beta _{2,14}X_1L_1 \nonumber \\&\quad + \beta _{2,15}X_1C. \end{aligned}$$
    This set or system of $T-d$ regression models for the repeated outcomes can be readily fitted jointly or simultaneously with, e.g., maximum likelihood estimation, in lavaan (Rosseel, 2012). The regression models specified in the illustration are also included as R model formulae in the online supplemental materials.
  - Predict the individual potential outcomes for $t=1,\ldots ,T-d$, under both exposure levels $x_t=0$ and $x_t=1$:
    A.6 $$\begin{aligned} \hat{m}_{t^\prime +d,t}(x_t,H_t)\equiv \hat{{\,\textrm{E}\,}}(R_{t^\prime +d, t+1}|X_t=x_t, H_t), \quad x_t=0,1. \end{aligned}$$
    The predictions can be computed using the jointly fitted models in the previous step, after setting the exposure levels to the respective hypothetical values of $x_t$ and retaining the observed values of pre-exposure covariates $H_t$, with the lavPredictY() function in lavaan.
  - Calculate the individual pseudo-outcomes for $t=1,\ldots ,T-d$:
    A.7 $$\begin{aligned} R_{t^\prime +d,t} = \dfrac{\delta \hat{\pi }_t m_{t^\prime +d,t}(1,H_t)+(1-\hat{\pi }_t) m_{t^\prime +d,t}(0,H_t)}{1+(\delta -1)\hat{\pi }_t}. \end{aligned}$$
5. For the outcome at time $t^\prime$, calculate for each individual, indexed by the superscript *i*, the following quantity:
  A.8 $$\begin{aligned} \hat{\phi }_{t^\prime }^{i} = \tilde{W}_{t^\prime }^{i} Y_{t^\prime }^{i} + \sum _{s=1}^{t^\prime } \tilde{W}_s^{i}V_s^{i} R_{t^\prime ,s}^{i}. \end{aligned}$$
  The estimator of the IPSICE for the outcome at time $t^\prime$ is then the sample average over all *N* individuals:
  A.9 $$\begin{aligned} \hat{{{\,\textrm{E}\,}}}(Y^{{\varvec{Q}}^\delta }_{t^\prime }) = \dfrac{1}{N} \sum _i \hat{\phi }_{t^\prime }^{i}. \end{aligned}$$
  Let $z_{1-\alpha /2}$ denote the $100(1-\alpha /2)$ percentile of a standard normal distribution; e.g., $z_{0.975} \approx 1.96$. An asymptotic Wald $100(1-\alpha )\%$ confidence interval (CI) can be constructed as:
  A.10 $$\begin{aligned} \hat{{{\,\textrm{E}\,}}}(Y^{{\varvec{Q}}^\delta }_{t^\prime }) \pm z_{1-\alpha /2}\hat{\sigma }_{t^\prime }(\delta ), \end{aligned}$$
  where $$\hat{\sigma }_{t^\prime }(\delta )=\sqrt{\dfrac{1}{N(N-1)} \sum _i \left\{ \hat{\phi }_{t^\prime }^{i} - \hat{{{\,\textrm{E}\,}}}(Y^{{\varvec{Q}}^\delta }_{t^\prime })\right\} ^2}$$.

To evaluate whether the average potential outcome under a posited value of $\delta$ is statistically significantly different from the average observed outcome, compare the CIs for the IPSICEs under $\delta \ne 1$ and $\delta =1$, respectively. If the endpoints of the CIs are disjointed, it indicates that shifting the propensity score odds for the recurring exposure by $\delta$ produces a change in the average outcome.

**Table 2:** Results of the simulation study comparing estimators of the average causal effects of a recurring exposure on $Y_1$ and $Y_2$

		Bias				ESE				RMSE			
		LR		IPSI		LR		IPSI		LR		IPSI	
*N*	$\alpha$	$Y_1$	$Y_2$	$Y_1$	$Y_2$	$Y_1$	$Y_2$	$Y_1$	$Y_2$	$Y_1$	$Y_2$	$Y_1$	$Y_2$
400	0	-0.00	0.57	-0.01	-0.06	0.19	0.25	0.33	2.03	0.19	0.62	0.33	2.03
6400	0	-0.00	0.56	0.00	-0.03	0.05	0.06	0.09	1.02	0.05	0.56	0.09	1.02
14,400	0	-0.00	0.56	-0.00	-0.01	0.03	0.04	0.06	0.46	0.03	0.56	0.06	0.46
400	2	0.01	0.61	0.01	-0.00	0.19	0.29	0.32	1.63	0.19	0.68	0.32	1.63
6400	2	0.00	0.62	0.00	-0.02	0.05	0.07	0.09	0.58	0.05	0.63	0.09	0.58
14,400	2	0.00	0.62	0.00	-0.01	0.03	0.05	0.06	0.53	0.03	0.62	0.06	0.53
400	4	0.00	0.66	-0.01	0.08	0.19	0.30	0.31	0.97	0.19	0.73	0.31	0.98
6400	4	0.00	0.66	0.00	0.02	0.05	0.08	0.09	0.77	0.05	0.66	0.09	0.77
14,400	4	-0.00	0.66	-0.00	0.01	0.03	0.05	0.06	0.40	0.03	0.66	0.06	0.40

*Note*. ESE = empirical standard error; RMSE = root mean squared error; LR = linear regression; IPSI = incremental propensity score intervention

Researchers should generally investigate the IPSICEs under different values for $\delta$. For example, because $\delta$ parame-trizes an odds ratio, an evenly spaced grid on the binary logarithm (Weisstein, 2024) scale may be $\Delta =\{2^{-2}, 2^{-1}, 2^{0}=1, 2^{1}, 2^{2}\}$. Then, for each posited value of $\delta \in \Delta$ in turn, repeat steps 2 through 5 to estimate the IPSICE under the given $\delta$. The estimates can then be plotted as a function of $\delta$ to visualize the pointwise trajectory as the IPSI parameterized by $\delta$ changes. Simultaneous $100(1\!-\!\alpha )\%$ confidence bands can be constructed by replacing $z_{1-\alpha /2}$ in (A.10) with an estimate of the $(1\!-\!\alpha )-$quantile of $\sup _{\delta \in \Delta } \sqrt{n}\dfrac{|\hat{{{\,\textrm{E}\,}}}(Y^{{\varvec{Q}}^\delta }_{t^\prime })-{{{\,\textrm{E}\,}}}(Y^{{\varvec{Q}}^\delta }_{t^\prime })|}{\hat{\sigma }_{t^\prime }(\delta )}$ using the multiplier bootstrap (Bonvini et al., 2023; Kennedy, 2019). There is a significant effect (at level $\alpha$) if the endpoints of the confidence bands for any two different $\delta \in \Delta$ are disjointed (Bonvini et al., 2023; Kennedy, 2019).

### Simulation study

We conducted a simulation study to empirically evaluate the operating characteristics of the IPSI estimator using lavaan. Each observed dataset was simulated using the following data-generating process:

$$\begin{aligned} U_1&\sim {\mathcal {N}}(0,1) \\ L_1&= 1.1 U_1 + \epsilon _{L1}, \quad \epsilon _{L1} \sim {\mathcal {N}}(0,1) \\ X_1&\sim \textrm{Bernoulli}\left( \dfrac{1}{1+\exp (-X_1^*)}\right) , \quad X_1^*= -2 L_1 \\ Y_1&= 1.4 U_1 + \epsilon _{Y1}, \quad \epsilon _{Y1} \sim {\mathcal {N}}(0,1) \\ U_2&\sim {\mathcal {N}}(0,1) \\ L_2&= 1.1 U_2 + 0.7 L_1 + X_1 \log (0.25+L_1^2) + \epsilon _{L2}, \quad \epsilon _{L2} \sim {\mathcal {N}}(0,1) \\ X_2&\sim \textrm{Bernoulli}\left( \dfrac{1}{1\!+\!\exp (-X_2^*)}\right) , \quad \!\!X_2^*\!=\! b_0 + \alpha L_2 X_1 \!+\! (-2 L_1)(1\!-\!X_1) \\ Y_2&= 1.4 U_2 + 0.9 Y_1 + \epsilon _{Y2}, \quad \epsilon _{Y2} \sim {\mathcal {N}}(0,1). \end{aligned}$$

The true ACEs of the recurring exposure on outcomes $Y_1$ and $Y_2$ were ${{\,\textrm{E}\,}}(Y_1^{1}-Y_1^{0})=0$ and ${{\,\textrm{E}\,}}(Y_2^{1,1}-Y_2^{0,0})=0$, respectively. The unobserved confounder $U_t$ was used to generate an association between $L_t$ and $Y_t$ within each time point $t=1,2$. The observed pre-exposure history $H_1=L_1$ for $t=1$ and $H_2=(L_1,L_2,X_1,Y_1)$ for $t=2$ sufficed for no unmeasured confounding in (5) to hold at each time point. To align the overall chances of the exposure across both time points, the value of the intercept $b_0$ was determined by minimizing the absolute difference between the sample marginal or average propensity scores, i.e., $|\Pr (X_2=1)-\Pr (X_1=1)|$. We used $\alpha$ to encode the strength of dependence of $X_2$ on $X_1$ for a recurring exposure. We considered three possible values of $\alpha$ and three sample sizes.

For the IPSI estimator, we specified the same propensity score as in the illustration. We assumed a logistic regression model for $X_t$ given all main and two-way interaction effects for the observed pre-exposure history in $H_t$; i.e., for $t=1$, (A.1) simplified to:

$$ {{\,\textrm{logit}\,}}\left\{ \Pr (X_1 =1 | H_1)\right\} = \alpha _{1,0} + \alpha _{1,1} L_1, $$

and for $$t=2$$,

$$\begin{aligned} {{\,\textrm{logit}\,}}\left\{ \Pr (X_2 =1 | H_2)\right\}&= \alpha _{2,0} + \alpha _{2,1} L_1 + \alpha _{2,2} L_2 + \alpha _{2,3} X_1 \\&\quad \!+ \alpha _{2,4} Y_1 + \alpha _{2,5} L_1L_2 + \alpha _{2,6} L_1X_1 \\&\quad \!+\! \alpha _{2,7} L_1Y_1 \!+\! \alpha _{2,8} X_1L_2 \!+\! \alpha _{2,9} Y_1L_2 \\&\quad \!+ \alpha _{2,10} X_1Y_1. \end{aligned}$$

For the outcome models, we assumed linear regression models with only main effects for $(X_t, H_t)$ for simplicity; i.e.,

$$\begin{aligned} {{\,\textrm{E}\,}}(Y_1 | X_1, H_1)&= \beta _{1,0} + \beta _{1,1}X_1 + \beta _{1,2}L_1, \\ {{\,\textrm{E}\,}}(Y_2 | X_2, H_2)&= \beta _{2,0} + \beta _{2,1}X_2 + \beta _{2,2}L_2 + \beta _{2,3}X_1 \\&\quad + \beta _{2,4}Y_1 + \beta _{2,5}L_1. \end{aligned}$$

We extracted estimators of the ACEs from those of the IPSIs by taking the difference in the latter for $\delta =2^{-9}$ and $\delta =2^{9}$ because these values corresponded to counterfactual scenarios where almost everyone is unexposed or exposed (Bonvini et al., 2023). For comparison, we fitted both outcome models jointly in lavaan, then used the maximum likelihood estimators of the linear regression coefficients $\beta _{1,1}$ and $\beta _{2,1}+\beta _{2,3}$ as estimators of the ACEs of *X* on $Y_1$ and $Y_2$, respectively. The results for 1000 replications under each setting are shown in Table 2. First, both linear regression and IPSI estimators of the ACE on $Y_1$ were unbiased in all settings. Second, the linear regression estimators of the ACE on $Y_2$ were biased in all settings. While the IPSI estimator suffered from finite sample biases, the biases decreased as the sample size increased. Third, the IPSI estimators of the ACE on $Y_2$ had smaller root mean squared errors than the linear regression estimators as the sample size increased. All the R scripts for replicating the results are available online (https://osf.io/8ksfx/).

## Footnotes

1 A fundamental assumption for well-defined individual potential outcomes is the *stable unit treatment value assumption* (SUTVA; Rubin (1986)). SUTVA comprises two components (Imbens & Rubin, 2015): (i) no hidden treatment variations and (ii) no treatment interference. The former requires the (absence of) treatment received must be the same for everyone, and the latter requires that an individual’s potential outcome must be unaffected by treatments received by other individuals.

2 In principle, IPSI can be implemented with other structural equation modeling software, such as Mplus (Muthén & Muthén, 1998-2017) and OpenMx (Boker et al., 2011), but the R functions we developed to estimate the IPSICE and generate the resulting plots were designed for compatibility with lavaan.

## Code availability

The R scripts implementing the estimation procedure are publicly available online at https://osf.io/8ksfx/.

## Declarations

### Open Practices Statements

The data used in the illustration is publicly available at https://doi.org/10.3886/ICPSR34598.v1. The R scripts implementing the estimation procedure are publicly available online at https://osf.io/8ksfx/.
